# Pharmacological Sequestration of Mitochondrial Calcium Uptake Protects Neurons Against Glutamate Excitotoxicity

**DOI:** 10.1007/s12035-018-1204-8

**Published:** 2018-07-14

**Authors:** Plamena R. Angelova, Darya Vinogradova, Margarita E. Neganova, Tatiana P. Serkova, Vladimir V. Sokolov, Sergey O. Bachurin, Elena F. Shevtsova, Andrey Y. Abramov

**Affiliations:** 1 Department of Molecular Neuroscience, UCL Institute of Neurology, Queen Square, London, WC1N 3BG UK; 2 Institute of Physiologically Active Compounds Russian Academy of Sciences, Chernogolovka, 142432 Russia

**Keywords:** Mitochondria, Calcium, Glutamate, Excitotoxicity, Neuron, Glutamate

## Abstract

Neuronal excitotoxicity which is induced by exposure to excessive extracellular glutamate is shown to be involved in neuronal cell death in acute brain injury and a number of neurological diseases. High concentration of glutamate induces calcium deregulation which results in mitochondrial calcium overload and mitochondrial depolarization that triggers the mechanism of cell death. Inhibition of mitochondrial calcium uptake could be potentially neuroprotective but complete inhibition of mitochondrial calcium uniporter could result in the loss of some physiological processes linked to Ca^2+^ in mitochondria. Here, we found that a novel compound, TG-2112x, can inhibit only the lower concentrations mitochondrial calcium uptake (induced by 100 nM-5 μM) but not the uptake induced by higher concentrations of calcium (10 μM and higher). This effect was not associated with changes in mitochondrial membrane potential and cellular respiration. However, a pre-treatment of neurons with TG-2112x protected the neurons against calcium overload upon application of toxic concentrations of glutamate. Thus, sequestration of mitochondrial calcium uptake protected the neurons against glutamate-induced mitochondrial depolarization and cell death. In our hands, TG-2112x was also protective against ionomycin-induced cell death. Hence, low rate mitochondrial calcium uptake plays an underestimated role in mitochondrial function, and its inhibition could protect neurons against calcium overload and cell death in glutamate excitotoxicity.

## Introduction

Glutamate is one of the major neurotransmitters in the brain. A number of conditions, which lead to exposure of neurons to excessive concentrations of glutamate, induce excitotoxic cell death [1]. Glutamate excitotoxicity is suggested to be a primary mechanism of acute brain injury, including brain trauma and stroke. Although there is a number of compounds shown to be protective against glutamate toxicity on primary neurons in culture, clinical trials data demonstrated unsatisfactory results for drugs designed to treat conditions incited by excitotoxicity [2].

Activation of glutamate receptors induces calcium influx into the cytosol of neurons where it is buffered by mitochondria. Calcium ATPases and ion exchangers on the plasma or ER membrane transport calcium out of the cytosol and restore basal calcium concentrations. Under pathological conditions, high concentration of glutamate induces delayed calcium deregulation and profound mitochondrial depolarization which is shown to be the trigger for neuronal cell death [3, 4]. Mitochondrial depolarization is calcium-dependent and can be prevented by specific inhibition of mitochondrial calcium uptake [5].

Mitochondria uptake calcium via specific electrogenic carrier named mitochondrial Ca^2+^ uniporter (MCU) [6]. Knockout of MCU is shown to be protective against excitotoxicity induced by NMDA [7]. Despite the recent developments of molecular tools to identify the molecular determinants of MCU, it is difficult to estimate the role of glutamate-induced mitochondrial depolarization and cell death for a number of reasons. On one side, Ruthenium Red and its active component Ru360, which have been used for inhibition of MCU for many years, specifically inhibit mitochondrial calcium uptake only in isolated mitochondria. However, Ruthenium Red is almost impermeable for cells and inhibits a large number of plasmalemmal ion channels; Ru360 is oxidizing fast and on a cellular level can be successfully used only for short-term experiments. On the other hand, mitochondrial calcium uptake can be restricted by mitochondrial depolarization [8], but it can induce bioenergetic failure that is enhancing the glutamate toxicity [9–12].

Here, we demonstrate that our newly synthesized compound TG-2112x protects neurons against calcium overload toxicity in the presence of ionomycin and against glutamate excitotoxicity by inhibition of glutamate-induced mitochondrial depolarization. The effect of TG-2112x on the mitochondrial membrane potential could be mainly explained by the limitation of mitochondrial calcium uptake by this compound. However, TG-2112x successfully inhibits mitochondrial calcium uptake induced only by low calcium concentration but it could not block Ca^2+^ influx induced by higher concentrations of calcium in the same way as RU360. We therefore suggest that TG-2112× can only partially inhibit MCU or, that is more likely, to block MCU-independent mitochondrial calcium uptake. Here, we find an importance of low calcium uptake in glutamate-induced mitochondrial depolarization and in the mechanism of cell death induced by excitotoxicity.

## Materials and Methods

### Cell Culture

Co-cultures of cortical or hippocampal neurons and astrocytes were prepared as described previously [13, 14] with modifications, from Sprague-Dawley rat pups 2–4 days post-partum (UCL breeding colony). Brain cortices or hippocampi were removed into ice-cold HBSS (Ca^2+^, Mg^2+^-free, Invitrogen, Paisley, UK). The tissue was minced and trypsinized (0.1% for 15 min at 37 °C), triturated and plated on poly-D-lysine-coated coverslips, and cultured in Neurobasal medium (Gibco-Invitrogen, Paisley, UK) supplemented with B-27 (Gibco-Invitrogen, Paisley, UK) and 2 mM L-glutamine. The cultures were maintained at 37 °C (5% CO_2_) and the media changed twice a week. Cells were used at 12–15 days in vitro.

Cerebellar granule cells (CGC) were prepared from the postnatal rats (7–8 days old, no sex separation) by the following procedure based on the generally accepted methods [8]. The pieces of cerebellum were digested with 0.25 mg/ml trypsin for 20 min at 37 °C. After washing, cells were dissociated by triturating. Following filtration through the nylon mesh, the cells were plated at a density of 2.5–5 × 10 cells per ml on poly(ethyleneimine)-coated 96-well plates (Corning) and maintained at 37 °C in a humidified incubator with 5% CO_2_/95% room air. The medium was composed of Eagle’s minimum essential medium: Dulbecco’s Modified Eagle’s Medium (Gibco)/Nutrient F-12 (Gibco) Ham 1:1 and fetal calf serum (10%) supplemented with 20 mM potassium chloride, 10 mM glucose, 2 mM glutamine, and 50 mg/ml gentamycin sulfate. Cytosine arabinoside (20 μg/ml) was added 24–48 h later to prevent the replication of non-neuronal cells.

#### Imaging [Ca^2+^]_c_ and Mitochondrial Membrane Potential

Hippocampal or cortical neurons were loaded for 30 min at room temperature with 5 μM fura-ff AM (Molecular probes) or 5 μM fura-2 AM (molecular probes) and 0.005% Pluronic in a HEPES-buffered salt solution (HBSS). For simultaneous measurement of [Ca^2+^]_c_ and mitochondrial membrane potential (ΔΨ_m_), Rh123 (1 μM, Molecular Probes) was added into the cultures during the last 15 min of the fura-2 or fura-ff loading period, and the cells were then washed 3–5 times before experiment.

Fluorescence measurements were obtained on an epifluorescence inverted microscope equipped with a × 20 fluorite objective. [Ca^2+^]_i_ and ∆ψ_m_ were monitored in single cells using excitation light provided by a Xenon arc lamp, the beam passing sequentially through 10-nm band pass filters centered at 340, 380, and 490 nm housed in computer-controlled filter wheel (Cairn Research, Kent, UK). Emitted fluorescence light was reflected through a 515-nm long-pass filter to a cooled CCD camera (Retiga, QImaging, Canada). All imaging data were collected and analyzed using software from Andor (Belfast, UK). The fura-2 or fura-ff data have not been calibrated in terms of [Ca^2+^]_i_ because of the uncertainty arising from the use of different calibration techniques. We have normalized the signals between resting level (set to 0) and a maximal signal (which correspond to full mitochondrial depolarization) generated in response to the protonophore FCCP (1 μM; set to 100%).

### Imaging of Mitochondrial Calcium

Confocal images were obtained using a Zeiss 710 CLSM using a × 40 oil immersion objective. The 488-nm Argon laser line was used to excite mitoGCaMP6f fluorescence which was measured at 505–550 nm. For Rhod-5 N measurements, the 563 nm excitation and 580–630 nm emission were used. All data presented were obtained from at least five coverslips and 2–3 different cell culture preparations.

### NADH and FAD Measurements

NADH autofluorescence was monitored using an epifluorescence inverted microscope equipped with a × 40 fluorite objective. Excitation light (350 nm) was provided by a Xenon arc lamp, the beam passing through a monochromator (Cairn Research, Kent, UK). Emitted fluorescence light was reflected through a 455-nm long-pass filter to a cooled CCD camera (Retiga, QImaging, Canada). Imaging data were collected and analyzed using software from Andor (Belfast, UK). FAD++ autofluorescence was monitored using a Zeiss 710 VIS CLSM and a × 40 objective. We used 454-nm laser for excitation with emission at 505–550 nm.

### Neuronal Toxicity Experiment

Cells were incubated with propidium iodide (PI; 20 μM) and Hoechst 33342 (4.5 μM; Molecular Probes, Eugene, OR). Viable cells exclude the red fluorescent PI (which can penetrate only in permeabilized cells) whereas Hoechst stains chromatin blue in all cells thus allowing dead cells to be quantified.

### Rat Brain Mitochondria Isolation

Rat brain non-synaptosomal mitochondria were isolated from Wistar strain male rats aged 3.5–4 months old (250–350 g), as previously described in [15]. Briefly, according to the regulations, the rats were anesthetized by carbon dioxide and killed by decapitation. The brain was quickly removed and homogenized in an ice-cold isolation buffer (IB), pH 7.4: 75 mM sucrose, 225 mM mannitol, 10 mM K-HEPES with addition of 0.5 mM EGTA, 0.5 mM EDTA, and 1 mg/ml BSA. The homogenate was centrifuged for 11 min at 1500×*g*. The pellet was homogenized in half the volume of the same buffer and centrifugation was repeated. The combined supernatants were centrifuged at 10500×*g* for 11 min. The resulting pellet was resuspended in 12% Percoll, layered to Percoll gradient (40–23–12%) and centrifuged at 30700×*g* at 4 °C for 15 min. The mitochondrial layer was collected and washed twice using centrifugation. The final pellet was re-suspended in the IB containing 0.02 mM EGTA.

The mitochondrial protein concentration was determined using a Biuret method with bovine serum albumin as a standard.

### Mitochondrial Permeability Transition

Ca^2+^-induced cyclosporine A-sensitive mitochondrial swelling was used to study the mitochondrial permeability transition. The mitochondrial swelling was determined by monitoring the absorbance at 620 nm using a Victor3 multi-well fluorescence plate reader (Perkin Elmer, Germany). The non-synaptosomal brain mitochondria (0.2 mg/mL) were incubated in a buffer, containing 75 mM sucrose, 225 mM mannitol, 10 mM K-HEPES (pH 7.4), 0.02 mM EGTA, 1 mM KH_2_PO_4_, 5 mM succinate, and 0, 5 ϻM rotenone. Compound or equal volumes of vehicle were added to mitochondria suspensions, and after 5 min, the mitochondrial permeability transition is induced by the 25 μM CaCl_2_ additions. The maximum swelling rate was calculated and normalized between rate of spontaneous swelling and maximum rate of CaCl_2_-induced swelling of control probe.

### Calcium Retention Capacity

Calcium accumulation and retention capacity was evaluated in the KCl-based medium supplemented with 100 nM Calcium Green-5 N (Molecular Probes) using a Victor3 multi-well fluorescence plate reader (Perkin Elmer, Germany) with ex/em = 506/535 nm. The brain mitochondria (0.2 mg/mL) were suspended in the KCl-based medium (120 mM KCl, 20 mM HEPES, 100 mM sucrose, 0.2 mM KH_2_PO_4_, 0.45 mM MgCl_2_, pH 7.2), containing substrates of respiratory chain (5 mM succinate with complex I inhibitor 1 μM rotenone, 5 mM glutamate/malate or 5 mM pyruvate/malate), 0.15 mM ADP, and 1 μg/mL oligomycin. All experiments were carried out in 96-well plates at 30 °C. The “Bolus mode” of calcium addition was used [15].

All experiments were replicated in at least three separate mitochondrial preparations. All figures are representative of at least four separate independent experiments.

### Seahorse Extracellular Flux Assay

Co-cultures of cortical neurons and glial cells were cultured on XF96 plates (Seahorse Bioscience-Agilent) at a density of 30,000–40,000 cells/well in neurobasal medium supplemented with B27, glutamine, glucose, and NaCl for 7–9 days. On the day of the assay, the cell culture medium was replaced with 150 μL/well of pre-warmed low-buffered medium (DMEM base medium supplemented with 25 mM glucose, 1 mM sodium pyruvate, 31 mM NaCl, 2 mM glutamine, pH 7.4) and the cells incubated at 37 °C for 30 min in a non-CO2 incubator. TG-2112x was prepared in DMSO and then diluted to the appropriate concentrations in the low-buffered medium. Oxygen consumption rates (OCR) and extracellular acidification rates (ECAR) of the neurons were measured at 37 °C using a Seahorse XF96 Extracellular Flux Analyzer (Seahorse Bioscience-Agilent; equipment of Center for Collective Use IPAC RAS - agreement N14.621.21.0008, ID RFMEFI62114X0008). Three baseline measurements of OCR were taken before injection of different concentrations of TG-2112x. Three readings were taken after each addition: different concentrations of TG-2112x, oligomycin (3 μM), FCCP (3 μM), rotenone (1 μM) with antimycin (1 μM). Basal OCR and changes in OCR upon addition of the mitochondrial modulators were recorded and calculated by the XF-96 software.

### Effect of TG-2112x on Ionomycin-Induced Toxicity

The 8–10 days CGC cells were incubated with a TG-2112x or an equal volume of the solvent (< 1% of the volume) and 3 μM ionomycin for 24 h. The cell viability was assessed as the dehydrogenase activity with the 3-(4,5-dimethylthiazol-2-yl)-2,5-diphenyltetrazolium bromide (MTT) assay. The measurements of absorbance were done using a Victor microplate reader (Perkin Elmer) at 570  nm.

No blinding was performed for all experiments.

### Statistical and Data Analysis

Statistical analysis and data analysis were performed using Origin 9 (Microcal Software Inc., Northampton, MA) software. Data was assessed for normality using the Shapiro-Wilk test. Statistical analysis was performed using unpaired two-tailed Student’s *t* test to analyze differences between two groups. A test for outliers was not conducted on the data. Results are expressed as means ± standard error of the mean (S.E.M.). Differences were considered to be significantly different if *p <* 0.05.

## Results

### Synthesis of TG-2112x

**Fig. 1:**
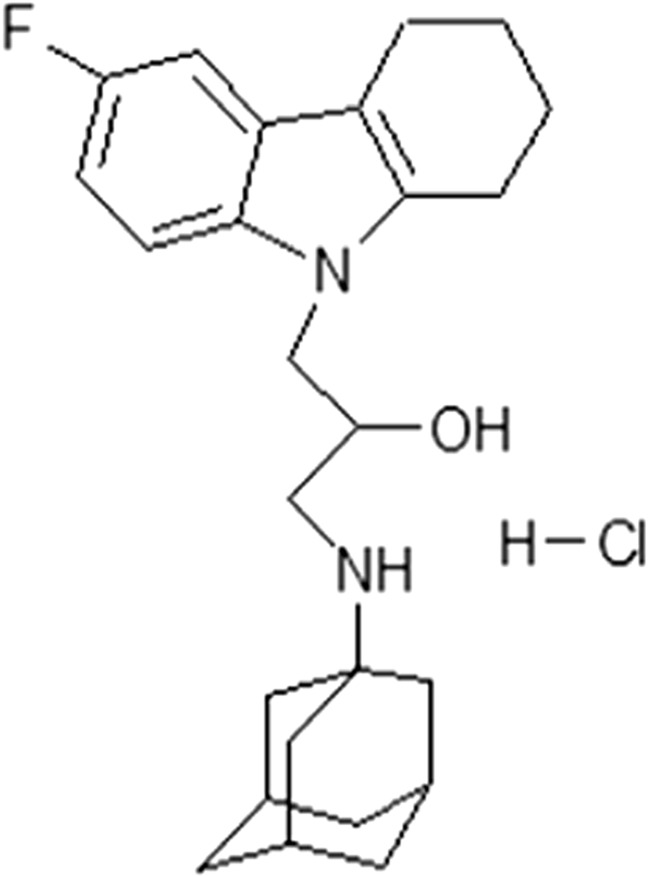
Structure of the compound TG-2112x

We synthesized our novel compound combining in one molecule two pharmacophore moieties of specific ligands whose biological targets are known to be involved in Alzheimer’s disease pathogenesis. In the assembly of hybrid polyfunctional molecules, we used aminoadamantane derivatives as the first pharmacophore and tetrahydrocarbazole derivatives as the second pharmacophore. In recent years, promising compounds for the design of disease-modifying drugs for the therapy of neurodegenerative diseases have been found among carbazole derivatives. In particular, these are aminotetrahydrocarbazoles that are able to stimulate neurogenesis and to stabilize the endoplasmic reticulum (ER) calcium homeostasis by attenuating the FAD-PS1-mediated exaggerated ER calcium release; they can improve the mitochondrial function measured by increased mitochondrial membrane potential and lower the Aβ peptide production by decreasing the cleavage of amyloid precursor protein (APP) by β-secretase, without notably affecting α- and γ-secretase cleavage activities. As a result of primary screening, the hit-compounds were revealed, which possess the properties of microtubules stabilizer, inhibitor of butyrylcholinesterase, and NMDA-receptors, and also prevent the calcium-induced mitochondrial swelling and protect the neuroblastoma SH-SY5Y against calcium overload [16]. Here, we study the possible mechanism of cytoprotective and mitoprotective effect of compound-leader TG-2112x (Fig.1).

### TG-2112x Protects Neurons Against Glutamate-Induced Mitochondrial Depolarization

**Fig. 2:**
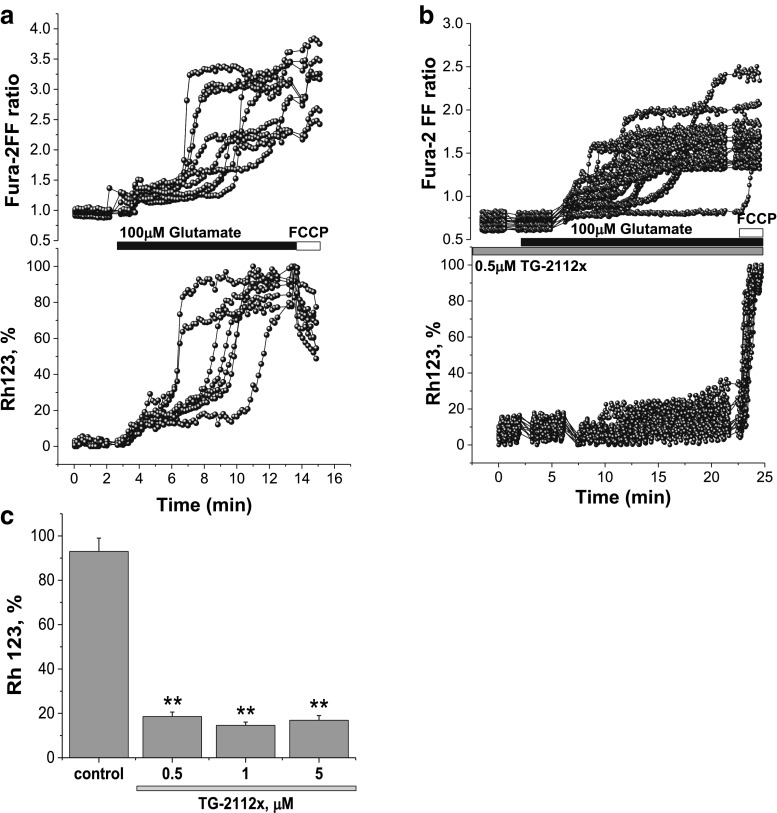
Effect of TG-2112x on [Ca^2+^]_c_ and mitochondrial membrane potential during 100 μM glutamate exposure. Simultaneous measurements of the changes in [Ca^2+^]_c_ (Fura-ff ratio) and Δψ_m_ (relative Rh 123 fluorescence) were made from single neurons. An increase in the Rh123 fluorescence reflects mitochondrial depolarization which corresponds to the effect of mitochondrial uncoupler FCCP (1 μM) which was applied at the end of this and subsequent experiments to reflect complete dissipation of Δψ_m_. **a**, **b**. illustration of the differences in the dynamics of [Ca^2+^]_c_ and Δψ_m_ in control (**a**) and in response to pre-incubation of the cells with 0.5 μM TG-2112x (**b**). **c** Summaries of the effects of the different concentration of TG-2112x on glutamate-induced mitochondrial depolarization (*n* = 88 neurons for control and 67, 59, and 89 cells for 0.5, 1, and 5 μM TG-2112x). **p < 0.001

**Fig. 3:**
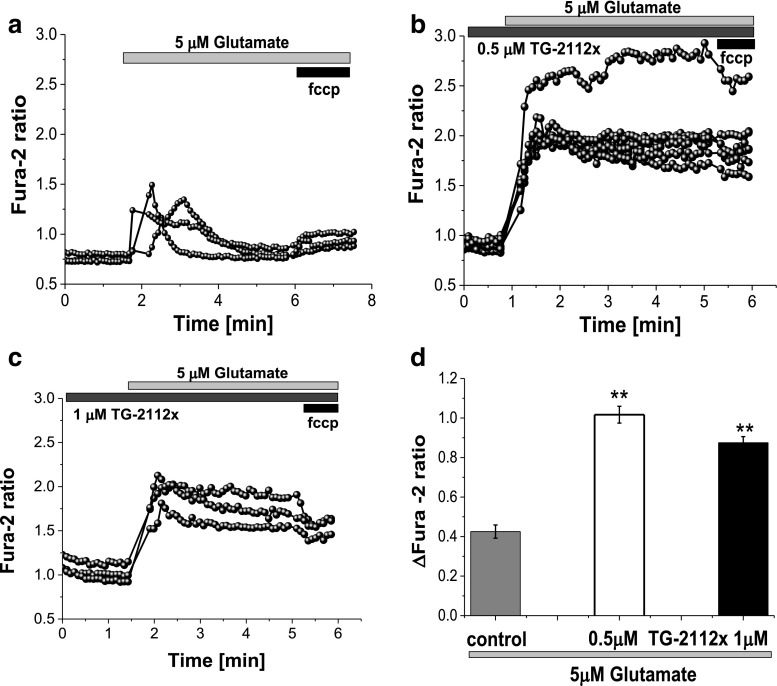
Effect of TG-2112 on [Ca^2+^]_c_ changes in neurons under application of physiological concentration of glutamate. Pre-incubation of cells with 0.5 μM (**b**) or 1 μM (**c**) TG-2112x increased the effect of 5 μM glutamate on [Ca^2+^]_c_ neurons compare to control (**a**). **c** Summary of the effects of TG-2112x on glutamate-induced calcium signal shown as a difference in Fura-2 ratio. (*n* = 67 cells- in control; *n* = 56 cells (0.5 μM TG-2112x); *n* = 76 cells (1 μM) **p < 0.001

In agreement with previously published data, we show that application of 100 μM glutamate to mature (≥ 12 days in vitro) hippocampal neurons induce an initial transient rise in [Ca^2+^]_c_ followed after a several minutes by a delayed increase to a plateau (Fig. 2a) [4, 5, 17]. Simultaneous measurements of calcium and mitochondrial potential showed that the delayed secondary increase in [Ca^2+^]_c_ is accompanied by a progressive and possibly complete loss of mitochondrial membrane potential (Fig. 2a, c). Pre-incubation of the cells with 0.5–1 μM TG-2112x did not change effect of 100 μM glutamate on primary or delayed increase of [Ca^2+^]_c_ of cortical neurons (Fig. 2b, c). However, in the majority of neurons, delayed calcium deregulation did not induce changes in ∆ψm (*n* = 67 out 88 neurons); in the rest of the cells, 100 μM glutamate induced only minor depolarization (from 93 ± 6% of Rh123 signal in control to 18.6 ± 2 in TG-2112 (0.5 μM) treated cells; *p* < 0.001; Fig. 2c). It has been accepted that the profound decrease of ∆ψm is dependent on Ca^2+^ influx, and this effect is abolished if glutamate is applied in the absence of extracellular Ca^2+^; moreover, it can be blocked specifically by inhibitor of mitochondrial calcium uniporter RU360 [5, 18]. Glutamate-induced neuronal loss requires mitochondrial calcium accumulation [18]. In order to test if TG-2112x changes calcium influx to cytosol in response to activation of glutamate receptors, we used small physiological concentrations of glutamate (5 μM) on the cortical co-culture loaded with fura-2. Glutamate induces transient peak followed by a recovery of [Ca^2+^]_c_ (Fig. 3a). Importantly, pre-incubation (5 min) of the cells with 0.5 or 1 μM TG-2112x significantly increased the [Ca^2+^]_c_ of neurons in response to glutamate (from 0.42 ± 0.03 (*n* = 67 cells) in control to 1.02 ± 0.04 (*n* = 56 cells; *p* < 0.001) and to 0.87 ± 0.03 (*n* = 76 cells; *p* < 0.001) for 0.5 μM and 1 μM TG-2112x; Fig. 3b, c, d). Thus, TG-2112x increases neuronal calcium responce to physiological concentration of glutamate that can suggest inhibition of Ca^2+^ efflux or blocking buffering of calcium by mitochondria.

### TG-2112x Inhibits Glutamate-Induced Mitochondrial Calcium Uptake in Neurons

**Fig. 4:**
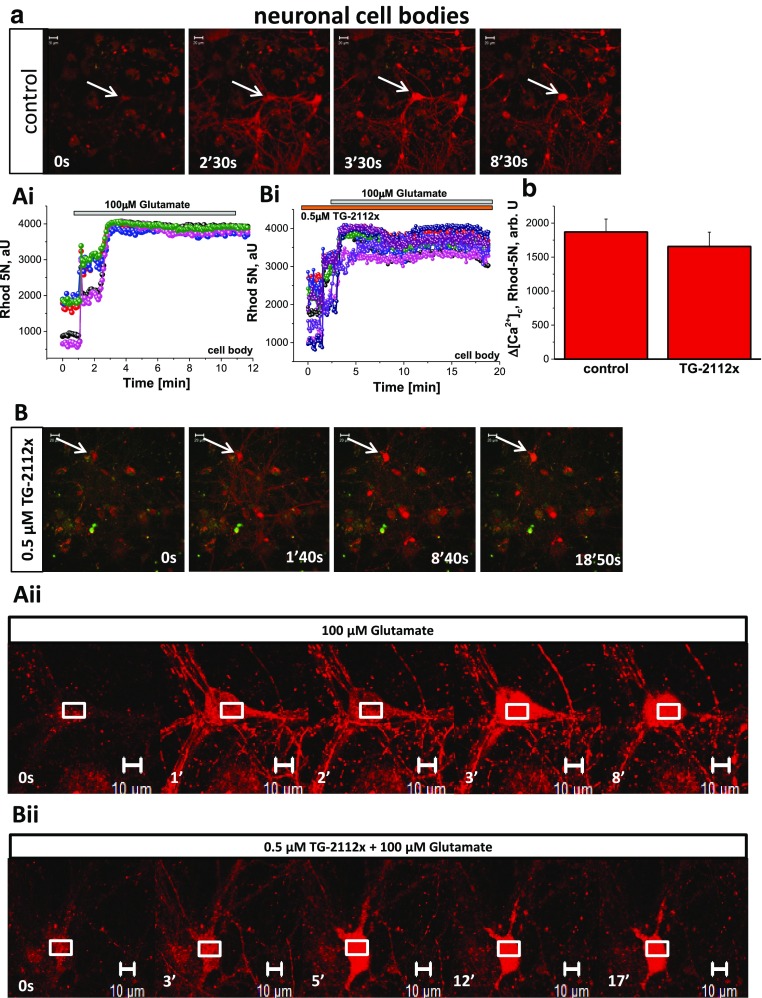
TG-2112x had no effect of the glutamate-induced mitochondrial calcium overload. Glutamate induced delayed calcium deregulation in control (**a**, **a**ii) and in TG-2112x (0.5 μM; **b**, **b**ii)-treated neurons measured with low affinity calcium indicator Rhod-5n. Effect 100 μM on mitochondrial calcium is impossible to detect in cell body due to DCD, but the effect of TG-2112x on glutamate-induced calcium uptake is easily detectable in processi (**a**ii, **b**ii). Traces represent individual cells (measured from cytosolic area **a**i) or mitochondria (**b**i). **c** Summary of the cytosolic responses to glutamate in control (*n* = 122 neurons) and treated with TG-2112x (0.5 μM; *n* = 99 neurons)

**Fig. 5:**
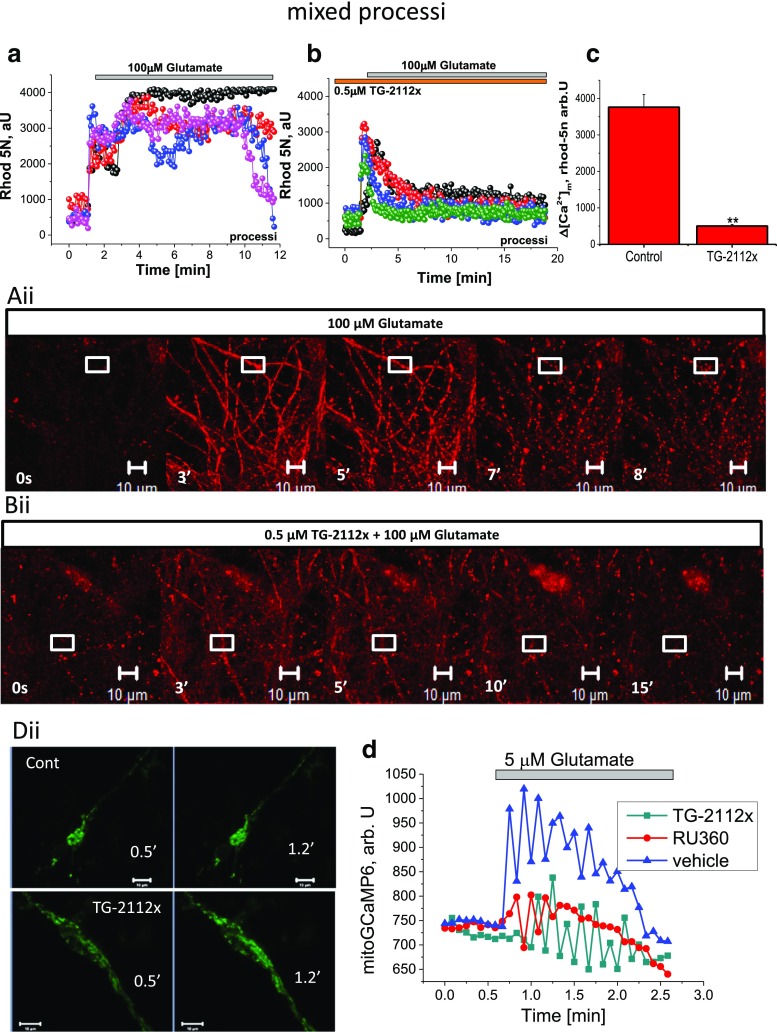
TG-2112x protects neurons against glutamate-induced mitochondrial calcium overload. TG-2112x (0.5 μM) blocks the calcium rise in majority of the mitochondria in the neuronal processi (**b**, **b**ii) compare to control neurons (**a**, **a**ii). Note that rise in rhod-5n signal in TG-2112x-treated neurons has recovered after an initial peak compared to the control neurons. The traces represent individual mitochondria from the processi. **c** Summary of the mitochondrial responses to glutamate in control (*n* = 122 neurons) and treated with TG-2112x (0.5 μM; *n* = 99). **p < 0.001. **d** (representative traces), **d**ii (representative images)—effect of 5 μM glutamate in control (*n* = 26 neurons), TG-2112x-treated cells (*n* = 35) and neurons treated with 10 μM Ru360 (*n* = 21 neuron) measured with genetically-encoded indicator for mitochondrial calcium MitoGCaMP6

In order to test the effect of TG-2112x on mitochondrial calcium transport at the time of application of toxic concentration of glutamate, we used low affinity calcium indicator rhod-5n, which is predominantly localized in mitochondria due to its negative charge. However, it is still present in the cytosol, that allows us to measure [Ca^2+^]_c_ changes, including the delayed calcium deregulation. Exposure of cortical neurons to 100 μM glutamate induced delayed calcium deregulation which can be seen in the cell body of the neurons (Fig. 4ai, bi). Mitochondrial signal which has appeared in the cell body has become impossible to separate from the rest of the signal due to high [Ca^2+^]_c_ in the cytosol of the neuronal body (Fig. 4a, b; aii, bii). However, mitochondria stay distinguishable in the processi even in the time of delayed calcium deregulation in cell body, and the rise in mitochondrial calcium ([Ca^2+^]_m_) can be easily measured in neuronal dendrites (Fig. 4aii, bii Fig. 5a, b). However, in agreement with the data obtained with fura-ff (Fig. 2a, b), pre-incubation of cortical neurons with TG-2112x (5 min) did not change the effect of 100 μM glutamate on [Ca^2+^]_c_ (Fig. 4ai, bi) but in contrast to control, it suppressed the rise in [Ca^2+^]_m_ that can be easily detected in the area of the processi (Fig. 4aii, bii; Fig. 5a, b). It should be noted that TG-2112x did not completely block mitochondrial calcium uptake in these cells, but protected against calcium overload. Thus, TG-2112x inhibits mitochondrial calcium uptake in glutamate-stimulated neurons.

Considering all difficulties in separation of the Rhod-5n signal in cytosol and mitochondria, we used genetically encoded indicator for mitochondrial Ca^2+^ MitoGCaMP6 (Fig. 5d, dii). Glutamate (5 μM) induced oscillation in MitoGCaMP6 fluorescence, which was not observed under resting conditions (Fig. 5d) which can be explained by the nature of mitochondrial uptake in these cells or by some properties of the indicator because it was observed in control and in experiments with inhibitors. Application of the 5 μM glutamate to TG-2112x treated neurons (0.5 μM for 20 min) induced significant decrease in mitochondrial calcium uptake (from 237 ± 29 arb. U to 86 ± 14 arb U, *n* = 26 cells and 35 cells for vehicle and TG-2112x; *p* < 0.01). Importantly, Tg-2112x changed also the shape of the signal (Fig. 5d). The effect of the TG-2112x was comparable with the pre-treatment of the neurons with Ru360 (10 μM for 20 min, changing the solution with fresh Ru360 to avoid oxidation of the compound; Fig. 5d). Application of 5 μM glutamate to neurons incubated with Ru360 significantly but not completely reduce calcium rise in mitochondria (from 237 ± 29 arb. U in control to 68 ± 16 arb. U, *n* = 21 neuron; *p* < 0.01).

### TG-2112x Inhibits Calcium-Induced Swelling of Isolated Rat Brain Mitochondria

In order to identify if TG-2112x has a direct action on mitochondrial calcium uptake, we study the effect of this compound on the calcium-induced mitochondrial swelling.

**Fig. 6:**
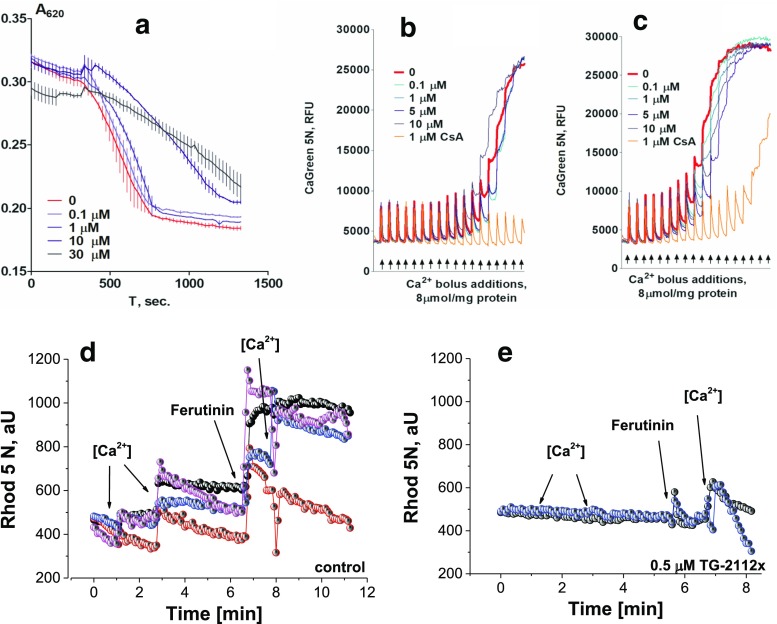
Effect of TG-2112x on the mitochondrial calcium uptake. TG-2112x inhibits calcium-induced swelling of isolated rat brain mitochondria (**a**). TG-2112x influence on the calcium retention capacity in isolated rat brain mitochondria with different respiratory substrates (**b** 5 mM glutamate/malate, **c** 5 mM succinate with complex I inhibitor 1 μM rotenone). Application of 100 nM-5 μM calcium to mitochondria in permeabilized neurons and astrocytes induced rise in [Ca^2+^]_m_ in control (**d**, *n* = 5 experiments), but not in TG-2112x (0.5 μM; *n* = 4 experiments)-treated mitochondria (**e**)

We found that TG-2112x attenuates the Ca^2+^-induced swelling of isolated rat brain mitochondria in a concentration-dependent manner with significant inhibition above concentration of 1 μM (Fig.6a). However, TG-2112x at concentration of 30 μM and above induces mitochondrial swelling without addition of calcium suggesting some membrane modifying effects of this compound at high concentrations.

### Effect of TG-2112x on Calcium Retention Capacity

Application of calcium to isolated mitochondria induces mitochondrial Ca^2+^ uptake and can be blocked by inhibitor of the calcium uniporter - Ru360. Incubation of mitochondria with TG-2112x did not block mitochondrial calcium uptake initiated by application of single or several boluses of CaCl_2_ (8 μmol/mg mitochondrial protein or 1.5 ÷ 2 μM) and moreover TG-2112x at 100 nM-10 μM increased mitochondrial calcium retention capacity with calcium additions in the “bolus mode” (Fig. 6c), and only at concentration above 5 μM, the decrease in CRC and the rate of calcium uptake were observed. It should be noted that effects of TG-2112x on mitochondrial calcium uptake were not dependent of the type of mitochondrial substrate used (Fig. 6b, c).

### TG-2112x Inhibits Calcium Uptake in Mitochondria of Permeabilized Neurons and Astrocytes

Another direct way to estimate the effect of TG-2112x on MCU is measurement of Ca^2+^ uptake in mitochondria of permeabilized cells. These experiments allow to directly measure the kinetics of Ca^2+^ uptake under application of variety of calcium concentrations in the pseudo-intracellular recording solution [19]. Additions of buffered calcium (0.5 and 1 μM, *n* = 5 experiments; Fig. 6d) increased mitochondrial calcium content. Application of the electrogenic calcium ionophore ferutinin (20 μM; [20, 21] induced further increase in [Ca^2+^]_m_. Application of the same calcium concentrations to the permeabilized neurons and astrocytes in the presence of TG-2112x (0.5 μM; *n* = 4 experiments) did not induced any increase in [Ca^2+^]_m_. Importantly, these mitochondria have been still potent, with maintained ∆ψm that allows electrogenic ionophores to work, and ferutinin induced increase in mitochondrial calcium. Moreover, further application of buffered calcium (1 μM) induced increase of [Ca^2+^]_m_ in TG-2112x-treated mitochondria (Fig. 6e). Thus, TG-2112x inhibits physiological influx into mitochondria (uniporter) while alternative transport, electrogenic calcium ionophore, is still able to produce increase of Ca^2+^ in these mitochondria.

### TG-2112x Does Not Induce Mitochondrial Dysfunction and Changes in ∆ψm

**Fig. 7:**
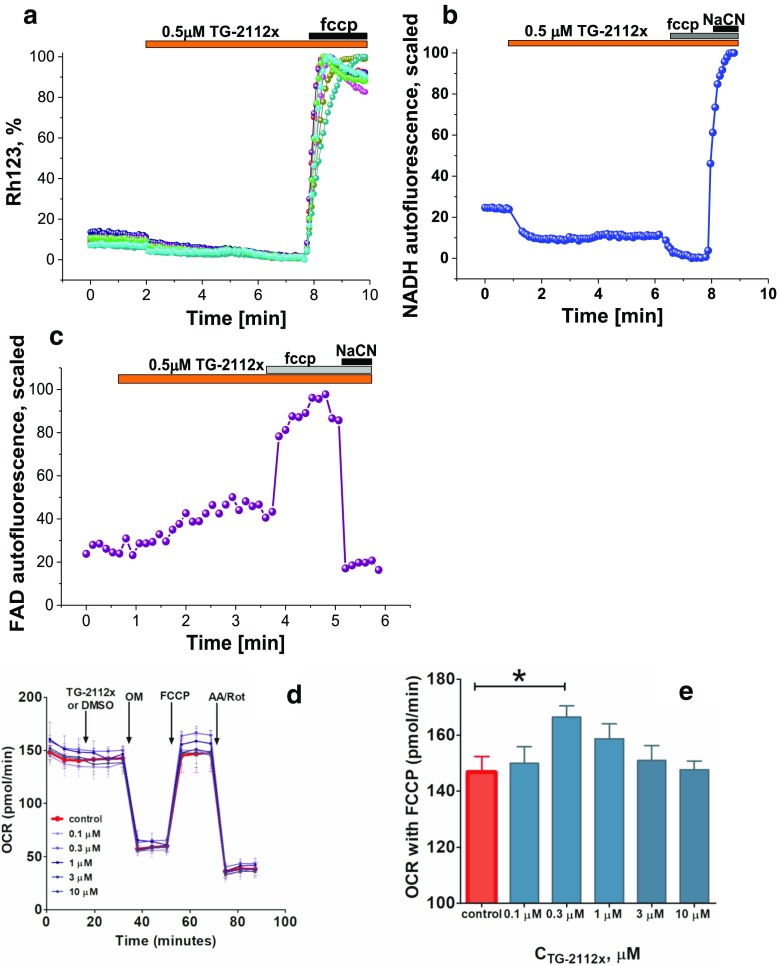
TG-2112x does not induce mitochondrial dysfunction. TG-2112x induced mild hyperpolarization of mitochondria (resulted in a decrease of Rh123 fluorescence; **a**; *n* = 4 experiments), decrease of mitochondrial NADH autofluorescence (**b**; *n* = 4 experiments), and increase in FAD++ fluorescence (**c**; *n* = 5 experiments) shown as representative traces from the single cells. TG-2112x did not influence the basal oxygen consumption rate (OCR), non-mitochondrial respiration of mixed cultures of cortical neurones, and glial cells (**d** lines represents the mean value traces of different experiments). OM oligomycin (3 μM), FCCP (3 μM), AA/Rot rotenone (1 μM) with antimycin (1 μM), but a tendency to an increased respiratory capacity in the presence of the mitochondrial uncoupler FCCP was observed (**e**; *n* = 5 of each experiments)

Complete and partial inhibition of mitochondrial calcium uptake could be induced by mitochondrial depolarization [22]. Using Rh123 as an indicator of mitochondrial membrane potential in intact neurons and astrocytes, we found that application of TG-2112x induces slow and progressive decrease (14 ± 1%; *n* = 4 experiments) in Rh123 fluorescence that corresponds to an increase of ∆ψm (complete depolarization by 1 μM FCCP at the end of the experiment produced the opposite effect on Rh123 fluorescence – Fig. 7a). Thus, the effect of TG-2112x on mitochondrial calcium transport is not due to an effect on ∆ψm.

In identifying the effect of the TG-2112x on the cellular bioenergetics, both oxygen consumption rates (OCR) and extracellular acidification rates (ECAR) of co-cultures of cortical neurones and astrocytes were measured in an XF96 extracellular flux analyzer. We have not detected any significant difference between the basal OCR, proton leak, ATP-linked respiration, non-mitochondrial oxygen consumption of control probe, and in the presence of 0.1 ÷ 10 μM of TG-2112x (Fig. 7d). However, a tendency to an increased respiratory capacity (FCCP application) was observed (Fig. 7e). In order to investigate effect of TG-2112x on mitochondrial respiration more specifically, we have measured NADH autofluorescence in co-cultures of primary neurons and astrocytes. Mitochondrial level of NADH is dependent on the activity complex I, and NADH autofluorescence correlates inversely with the respiratory chain activity. In order to identify mitochondrial NADH (which need to be separated from cytosolic NADH and NADPH autofluorescence), we added FCCP (1 μM) to maximize mitochondrial respiration that lead minimal level of NADH in mitochondria (taken as 0%), then added NaCN (1 mM) to inhibit mitochondrial respiration and therefore maximize the mitochondrial NADH (100%) (Fig. 7b). We have found that application of TG-2112x activates consumption of NADH in mitochondria that suggests activation of complex I-related respiration (Fig. 7b). Activity of complex II can be assessed by measuring autofluorescence of FAD^++^ considering that FAD(H_2_) is the complex II substrate. FAD^++^ redox level was estimated after application of FCCP (100%) and NaCN (taken as 0%). TG-2112x (0.5–1 μM) is increased autofluorescence of mitochondrial FAD that suggests activation of the mitochondrial complex II-related respiration in intact neurons and astrocytes (*n* = 5 experiments; Fig. 7c).

Thus, TG-2112x in concentrations which inhibit mitochondrial calcium uptake, stimulates respiration and increases mitochondrial membrane potential.

### TG-2112x Protects Neurons Against Glutamate- and Ionomycin-Induced Cell Death

**Fig. 8:**
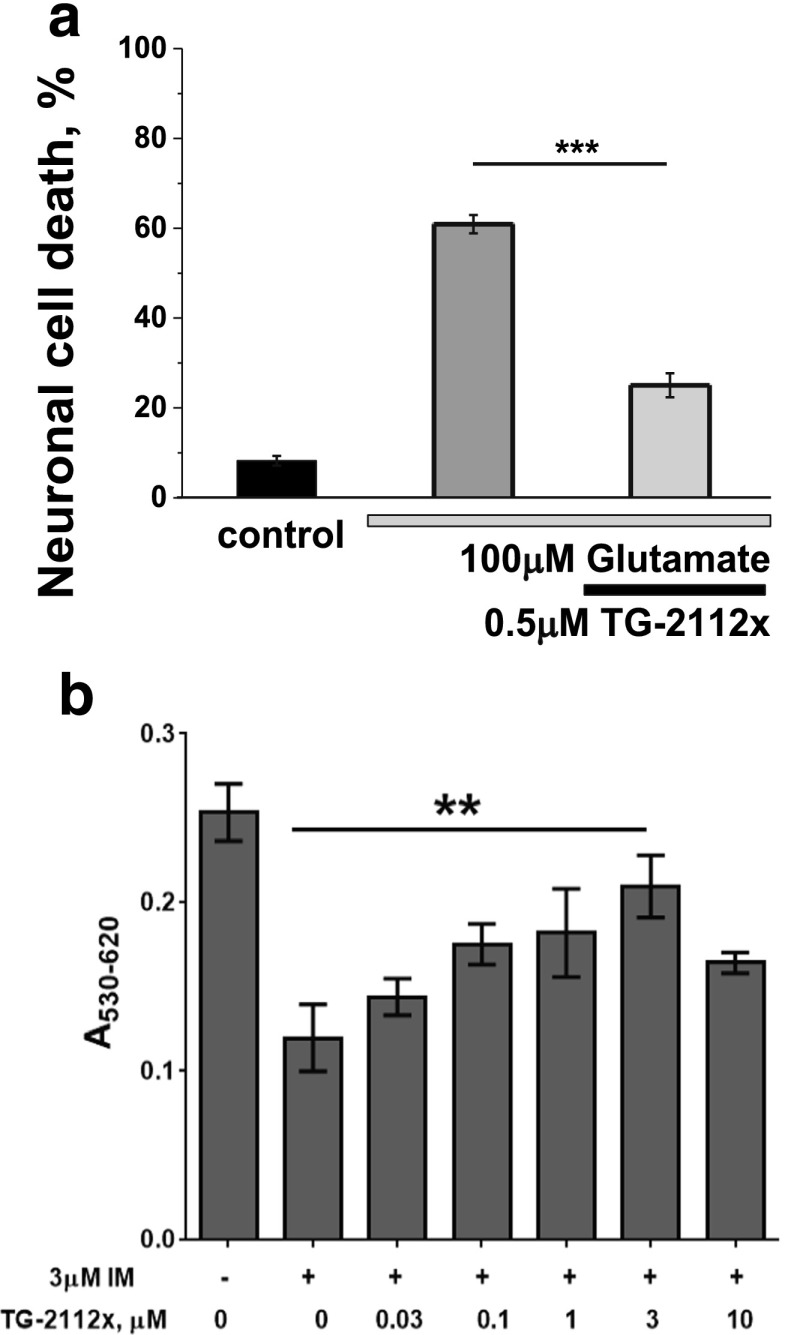
TG-2112x protects neurons against calcium-induced cell death. Pre-incubation of the cortical neurons with 0.5 μM TG2112x protects cells against cell death, induced by 100 μM glutamate (**a**). TG-2112x dose-dependently protects cerebellar granular cells against ionomycin-induced neuronal death (**b**), *n* = 4 of each experiments; **p < 0.001; ***p < 0.0001

We then examined the effect of 15-min exposure of cultures to 100 μM glutamate on cell viability (estimated after 24 h) and found that remarkably, 60.9 ± 2.03% of neurons died in this period (Fig. 8a, *n* = 4 experiments). Pre-incubation with 0.5 μM TG-2112x reduced cell death of cortical neurons to 25 ± 2.6%, *p* < 0.001; *n* = 4 experiments; Fig. 8a). Thus, suppression of mitochondrial calcium uptake by TG-2112x protects neurons against mitochondrial depolarization and cell death. Importantly, TG-2112x was protective not specifically against glutamate excitotoxicity but against calcium-induced cell death. Thus, treatment of primary culture of cerebellar granular cells with 3 μM ionomycin for 24 h induced cell death in ~ 50% cells (Fig. 8b). Pre-treatment of the cells with TG-2112x significantly protected them from glutamate-induced cell death, yielding maximal effect at concentrations of 1 and 3 μM.

## Discussion

The data from experiments described here suggest that (a) our novel compound TG-2112x protects neurons against neuronal cell death induced by toxic concentrations of glutamate; (b) the protective effect is not based on the inhibition of glutamate receptors—neurons exhibited similar to control delayed calcium deregulation; (c) TG-2112x blocked glutamate-induced mitochondrial depolarization in majority of the cells; and (d) the effect of TG-2112x on mitochondrial membrane potential and neuronal cell survival can be explained by the sequestration of the mitochondrial calcium that inhibits (more likely by blocking of low concentration calcium uptake) mitochondrial calcium overload.

The synchrony of mitochondrial depolarization and the calcium deregulation has suggested that these two processes must be interdependent and both essential in the mechanism of glutamate-induced cell death [23–25]. Here, we show that despite that first stage of mitochondrial depolarization is not linked to opening of mitochondrial permeability transition pore and trigger of cell death [5, 26]; the loss of ∆ψm is essential point in the mechanism of cell death. Delayed calcium deregulation for long time was associated as an initial point of the neuronal loss under glutamate exposure [27]. Here, we found that neurons could survive even after prolonged delayed calcium deregulation if their mitochondria are not depolarized. Although mitochondrial calcium uptake has been suggested as initial step in glutamate excitotoxicity [18] and mitochondrial calcium uptake was linked to mitochondrial depolarization [5], the direct protection of the neurons against high doses of glutamate by inhibition of mitochondrial calcium uptake is shown here.

There are number of ways to protect neurons against excitotoxicity—inhibition of the NMDA receptors, by stimulation of active calcium transport (Ca^2+^ ATPases) or by limitation of the NMDA receptors activation by neuromodulators [13, 28, 29]. Unfortunately, these strategies are limited by inhibition of the important physiological functions in the neurons under normal conditions. Sequestration, but not full inhibition of mitochondrial calcium uptake by TG-2112x, could be beneficial in the number of diseases with calcium-induced pathology including stroke, Alzheimer’s disease, and epilepsy [16, 30, 31]. Considering the modification of calcium signaling by alpha-synuclein [32] and inhibition of mitochondrial calcium efflux in mitochondria of neurons with familial forms of Parkinson’s disease, limitation of calcium uptake by TG-2112x would be also cell protective [33–35].

Application of TG-2112x in our experiments allow us to point one of the important questions in basic calcium studies, i.e., molecular inhibition of MCU has no effect on low concentration calcium uptake [36]. Effect of TG-2112x on mitochondrial calcium uptake which we observed here could be induced not by inhibition of MCU but exclusively on the system of low calcium uptake. This low concentration calcium uptake is very important considering the fact that concentration of Ca^2+^ in cytosol in physiology usually does not exceed 1 μM and under conditions of pathology (such as glutamate excitotoxicity) reaches 5–7 μM that is much lower than the threshold for MCU [18]. Protective properties of TG-2112x strongly suggest an important role of low concentration mitochondrial calcium uptake in physiology and pathology.

The mechanism of the low concentration calcium uptake could be solely dependent on one transporter or can have a multiple players. Thus, we cannot exclude the action of MCU in high affinity mode which is not affected by Ru360. However, this is a less likely mechanism considering low calcium uptake in MCU deficiency [35]. We also can exclude possible effect of TG-2112x on calcium precipitation with phosphate in mitochondria because this process requires higher Ca^2+^ concentration [37]. The number of the natural compounds could play the role of mitochondrial calcium ionophore [38, 39] such as prostaglandin [40], polyhydroxybutyrate, or polyphosphate [41, 42] and many others. We cannot exclude effect of TG-2112x on biochemical pathways which produce these potential modulators of mitochondrial calcium uptake.

## Abbreviations

ER: Endoplasmic reticulum
MCU: Mitochondrial Ca2+ uniporter
CGC: Cerebellar granule cells
HBSS: HEPES-buffered salt solution
∆ψm: Mitochondrial membrane potential
Rh123: Rhodamine 123
PI: Propidium iodide
OCR: Oxygen consumption rates
ECAR: Extracellular acidification rates
CRC: Calcium retention capacity
